# Metal-loaded Zeolite Remediation of Soils Contaminated with Pandrug-resistant *Acinetobacter Baumannii*

**DOI:** 10.2478/aiht-2020-71-3327

**Published:** 2020-06-29

**Authors:** Jasna Hrenović, Svjetlana Dekić, Jelena Dikić, Snježana Kazazić, Goran Durn, Nevenka Rajić

**Affiliations:** 1 University of Zagreb Faculty of Science, Department of Biology, Zagreb, Croatia; 2 University of Belgrade, Innovation Centre of the Faculty of Technology and Metallurgy, Belgrade, Serbia; 3 Ruđer Bošković Institute, Division of Physical Chemistry, Zagreb, Croatia; 4 University of Zagreb Faculty of Mining, Geology and Petroleum Engineering, Zagreb, Croatia; 5 University of Belgrade Faculty of Technology and Metallurgy, Belgrade, Serbia

**Keywords:** copper, environment, natural zeolite, pathogens, public health, silver, bakar, okoliš, patogeni, prirodni zeolit, srebro, zdravstvena zaštita

## Abstract

Due to the development of resistance to antimicrobial agents, bacterium *Acinetobacter baumannii* is nowadays a leading cause of nosocomial outbreaks. Clinically relevant *A. baumannii* outside hospital settings including natural soils affected by human waste represents a public-health risk for humans and animals. The aim of this study was to investigate the potential of metal-loaded zeolites to eliminate viable *A. baumannii* from artificially contaminated natural soils. *A. baumannii* isolate was subjected to the activity of natural zeolitised tuff (NZ) and Cu-modified (CuNZ) or Ag-modified zeolite (AgNZ) in wet, slightly acidic *terra rossa* and slightly alkaline red palaeosol. *A. baumannii* survived in *terra rossa* and red palaeosol supplemented with 1 wt% of NZ for seven days and four months, respectively. The addition of 1 wt% of CuNZ to *terra rossa* and red palaeosol shortened the survival of *A. baumannii* to three and 14 days, respectively. The addition of 0.1 wt% of AgNZ to both soils resulted in complete removal of viable *A. baumannii* within 1 h of contact, while the total native heterotrophic bacterial counts remained high. Since AgNZ is prepared with a simple modification of cost-effective and environmentally friendly natural zeolite, it is a promising material for the remediation of soils contaminated with pandrug-resistant *A. baumannii*.

## Abstract (HR)

Bakterija *Acinetobacter baumannii* u današnje je vrijeme vodeći uzročnik bolničkih infekcija, ponajprije zbog brzoga razvoja otpornosti te bakterije na antimikrobne lijekove. Klinički značajni *A. baumannii* izvan bolničkoga okružja, kao što su prirodna tla pod utjecajem ljudskoga otpada, javnozdravstveni su rizik za ljude i životinje. Cilj ovoga rada bio je istražiti potencijal zeolita modificiranih metalima za uklanjanje vijabilnih *A. baumannii* iz umjetno zagađenih prirodnih tala. Izolat *A. baumannii* u vlažnoj blago kiseloj terra rossa (pH 5,40) i blago lužnatom crvenom paleotlu (pH 8,43) podvrgnut je djelovanju prirodnoga zeolitnoga tufa (NZ) te NZ-a modificiranoga bakrom (CuNZ) ili srebrom (AgNZ). U slučaju dodatka 1 wt% NZ-a, *A. baumannii* je preživio u *terra rossa* sedam dana, a u crvenom paleotlu čak četiri mjeseca. Dodatak 1 wt% CuNZ-a u tla skratio je preživljavanje *A. baumannii* na tri dana u *terra rossa* i 14 dana u crvenom paleotlu. Dodatak samo 0,1 wt% AgNZ-a u tla rezultirao je kompletnim uklanjanjem vijabilnih *A. baumannii* tijekom jednoga sata kontakta, pri čemu je brojnost ukupnih heterotrofnih bakterija, prirodno prisutnih u tlu, ostala visoka. Čestice AgNZ-a pripremaju se jednostavnom modifikacijom jeftinoga i okolišno prihvatljivoga prirodnoga zeolita. Mala koncentracija (1 g AgNZ-a po 1 kg tla) mogla bi se jednostavno raspršiti po tlu koje je zagađeno bakterijom *A. baumannii*. Stoga je AgNZ obećavajući materijal za remedijaciju tala zagađenih tom bakterijom koja je otporna na sve dostupne antibiotike.

*Acinetobacter baumannii* is a neutrophilic, aerobic, Gram-negative, non-sporulating bacterium (1) that has no minimal infectious dose and is capable of developing pandrug resistance (resistance to all antimicrobial agents). This is why *A. baumannii* is an emergent opportunistic pathogen causing nosocomial infections worldwide (2, 3, 4). Infections have also been evidenced outside hospital environments (5), but the source for these community-acquired infections has not yet been determined beyond doubt.

However, soil has scarcely been investigated as a possible source of *A. baumannii*. One *A. baumannii* strain similar to a clinical isolate was reported in palaeosol contaminated with illegally disposed human solid waste (6). Three drug-resistant isolates of *A. baumannii* were recovered from technosol developed at one dumpsite (7). However, judging by a study of *A. baumannii* in pristine soils (8), this pathogen is not native to uncontaminated soils, which clearly points to contamination (leaching) from human solid waste.

*A. baumannii* can survive in a wide moisture, temperature, and pH range (9, 10, 11), which suggests long-term persistence in soil. In a water suspension of strongly acidic palaeosol, *A. baumannii* survived for one day (6), while in a pH-neutral technosol it survived for 58 days (7).

As drug-resistant *A. baumannii* in soil poses a public-health risk for humans and can also affect animals (12), we need to look for ways to remove this pathogen from contaminated soils. One of the ways to do that could be to use transition metal-containing natural zeolites due to their antibacterial properties. They have an advantage over free metal cations with antibacterial activity, since the aluminosilicate lattice of zeolites slowly releases metal cations into surroundings (13, 14). However, experiments with zeolites were carried out in physiological solution at 36 °C and are not applicable to the conditions in soil. To address that shortcoming, we investigated the antibacterial potential of metal-loaded zeolites directly in contaminated natural soils.

## Materials and methods

### A. baumannii *isolate properties*

The *A. baumannii* isolate (named EF7) chosen for the experiments was recovered from the effluent of a secondary wastewater treatment plant and deposited at the University of Zagreb Faculty of Science. This isolate is highly related to clinical isolates and is resistant to all tested antibiotics, which classifies it as pandrug-resistant (15, 16).

### Soil sampling and characterisation

Based on results of a previous investigation (17), we used two soil samples representative of slightly acidic and alkaline soil from Istria, Croatia – *terra rossa* and red palaeosol from Cretaceous limestone – dug from the upper 30 cm of 50 cm deep pits. Samples were aseptically collected in sterile plastic bags and analysed in the laboratory within 24 h of collection.

Soil pH value was measured with a WTWSenTix81 electrode (WTW, Weilheim, Germany) after triplicate suspension in distilled water (1:2.5 wt/v). The procedures for chemical (ICP-ES/MS following a lithium borate fusion and dilute nitric digestion) and mineralogical analyses (X-ray powder diffraction of <2 mm and <2 μm fractions) were described in detail in Durn et al. (18).

### Preparation of metal-loaded zeolites

Natural zeolitised tuff (NZ) was obtained from a Zlatokop sedimentary deposit in Serbia and consisted of 73 % clinoptilolite, 14 % plagioclase, and 13 % quartz (wt). To load NZ with metals, we used the ion-exchange procedure described in our earlier study (14). Briefly, it consisted of the following steps: 1) conversion of NZ into Na-enriched form (NaNZ) by treating NZ with 2 mol/L of NaCl solution at 25 °C for 48 h; 2) treatment of NaNZ with 6 mmol/L of Cu(NO_3_)_2_ or AgNO_3_ solution in a water bath at 25 °C for 24 h; and 3) recovering of the metal-containing products (CuNZ and AgNZ) by filtration.

### Leaching test

The leaching of cations from zeolites to soil samples is technically hard to track. Moreover, our previous investigation (13) showed negligible leaching of Cu^2+^ from CuNZ into the water medium. This is why we investigated only Ag^+^ leaching in this study. One gram of red palaeosol was supplemented with 1 mg (0.1 wt%) of AgNZ and added to 100 mL of autoclaved commercially available spring water and left in the dark at 25 °C for 1 h. The solid was separated from water by filtration through 0.45 μm syringe filters and the concentration of the leached Ag determined in water solution by atomic absorption spectrophotometer (AAS Varian, Spectra AA 55b, Agilent Technologies, Santa Clara, CA, USA).

### Experimental setup and bacterial counting

The *A. baumannii* isolate was grown on CHROMagar Acinetobacter supplemented with CR102 (CHROMagar, Paris, France) and 15 mg/L of cefsulodin (Sigma-Aldrich, St. Louis, MO, USA) at 36 °C for 24 h. The biomass was suspended in 300 mL of autoclaved commercial spring water. Based on preliminary experiments, this suspension was used to adjust soil moisture to their maximum water-holding capacity of 30 wt%.

Both soils were distributed in 100 g quadruplicate samples to laboratory glasses. *A. baumannii* suspension was added gradually by mixing it in with a sterile spatula. The content of eight glasses was mixed with 1 g of NZ, CuNZ, or AgNZ or 0.1 g of AgNZ. These experimental glasses were covered with parafilm and incubated in the dark at 25 °C until all viable *A. baumannii* disappeared. Soil moisture was kept constant (30 wt%) in all systems by adding autoclaved spring water as needed, which was determined by weekly gravimetrical measurements of soil moisture (by drying soil at 105 °C to constant weight) in a system supplemented with NZ.

**Figure 1:**
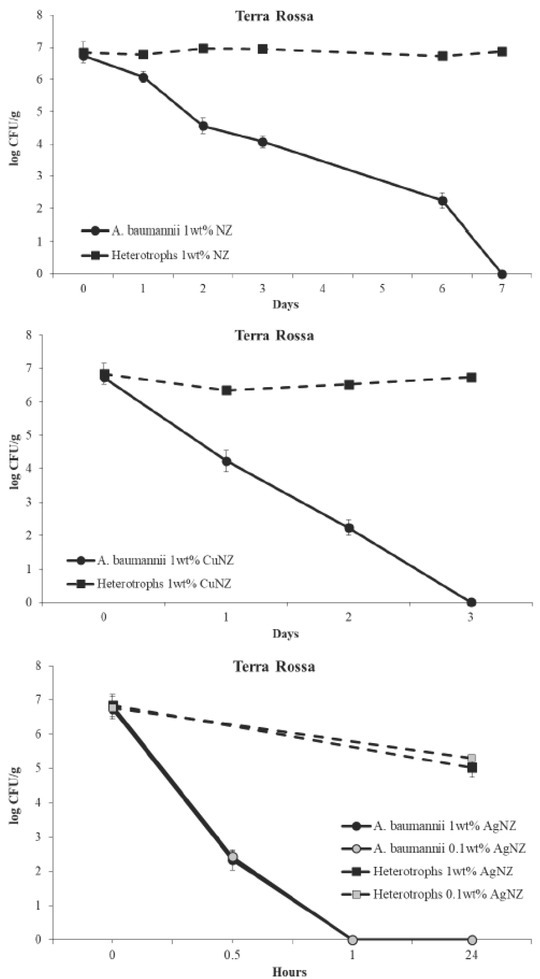
Removal of *A. baumannii* from slightly acidic *terra rossa* (pH 5.40) supplemented with 1 wt% of natural zeolitised tuff (NZ) or 1 wt% of Cu-loaded NZ (CuNZ), or 1 or 0.1 wt% of Ag-loaded NZ (AgNZ). Bacterial counts represent averages of triplicate counts ± standard deviation. The detection limit was 1 CFU/g

**Figure 2:**
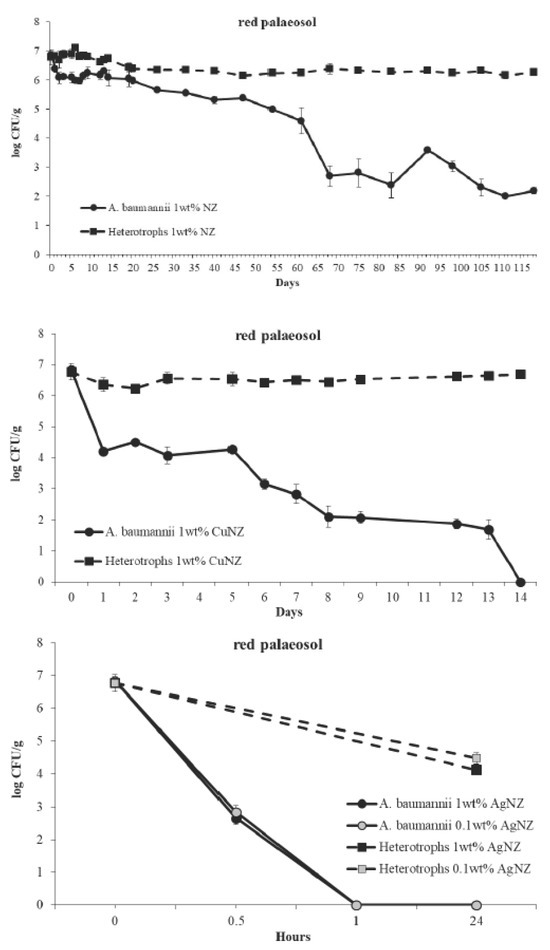
Removal of *A. baumannii* from slightly alkaline red palaeosol (pH 8.43) supplemented with 1 wt% of natural zeolitised tuff (NZ) or 1 wt% of Cu-loaded NZ (CuNZ), or 1 or 0.1 wt% of Ag-loaded NZ (AgNZ). Bacterial counts represent averages of triplicate counts ± standard deviation. The detection limit was 1 CFU/g

At the beginning of the experiment and at specified time points (Figures 1 and 2), the soil samples were mixed well and subsamples taken for bacteriological analysis in triplicate. One gram of wet soil was suspended in sterile physiological solution by vortexing at 45 Hz for 3 min and then passed through sterile membrane filters directly when bacterial concentrations were low or diluted decimally and inoculated on bacteriological media when the concentrations were sufficient for dilution.

Total native heterotrophic bacteria were counted on nutrient agar (Biolife, Milan, Italy) after incubation at 22 °C for 72 h. *A. baumannii* was cultivated on selective CHROMagar Acinetobacter medium supplemented with CR102 and cefsulodin after incubation at 36 °C for 48 h. This medium, intended for the cultivation of carbapenem-resistant bacteria, allowed for selective growth of carbapenem-resistant *A. baumannii*. This medium allows development of other Gram-negative bacteria resistant to β-lactam antibiotics (carbapenems and cephalosporins), but the large, circular, convex, smooth, red colonies of *A. baumannii* with a paler central area are clearly distinct from other bacterial species (19). Even so, we did not notice any interference of other bacteria. Native bacteria did not hinder *A. baumannii* counting.

Bacterial colonies were identified on whole cells with matrix-assisted laser desorption/ionisation – time of flight mass spectrometry (MALDI-TOF MS, software version 3.0, Microflex LT, Bruker Daltonics, Billerica, MA, USA). Randomly chosen colonies developed on CHROMagar Acinetobacter were confirmed as *A. baumannii*. Randomly chosen, morphologically different colonies of heterotrophic bacteria developed on nutrient agar (four plates per system) were also identified.

*A. baumannii* and total heterotrophic bacterial counts were expressed as log colony forming units (CFU) per one g of wet soil. The drop in *A. baumannii* count was calculated at the end of the experiments by subtracting bacterial count at the end from the baseline count [log CFU/g(start) - log CFU/g(end)].

### Statistical analysis

Statistical significance (p<0.05) of the difference between the baseline and end bacterial counts was established with Student’s *t-*test for independent variables using Statistica 13.3 (TIBCO Software, Inc., Palo Alto, CA, USA).

## Results and discussion

### Characteristics of soils and modified zeolites

*Terra rossa* was slightly acidic (pH 5.40±0.21), and red palaeosol slightly alkaline (pH 8.43±0.14). Both soils were dominated by quartz and clay minerals (illitic material and mica, kaolinite and mixed-layer clay minerals), followed by plagioclase, K feldspar, haematite, and goethite. Unlike *terra rossa*, red palaeosol also contained chlorite, 14 Å clay minerals, dolomite and calcite. *Terra rossa* and red palaeosol had similar chemical composition of both major oxides and trace elements. The only difference was the higher content of MgO and CaO in red palaeosol, which corresponds to its mineral composition (presence of calcite and dolomite) and slight alkalinity.

The samples of modified zeolites contained similar amounts of metal cations per gram of dry sample: 0.37 mmol Cu^2+^ (CuNZ) or 0.50 mmol Ag^+^ (AgNZ).

### *Remediation of* terra rossa *contaminated with* A. baumannii

In *terra rossa* supplemented with 1 wt% of NZ (Figure 1) *A. baumannii* survived for seven days, when its count dropped from of the baseline of 6.7 log CFU/g to below the detection limit (1 CFU/g). No statistically significant drop was observed for the native heterotrophic bacteria over these seven days. As NZ does not exhibit antimicrobial activity (14), this drop in *A. baumannii* count can be attributed to soil acidity, to which *A. baumannii* as a neutrophilic species is not adapted.

The addition of 1 wt% CuNZ (Figure 1) significantly accelerated the removal of *A. baumannii* to only three days of contact, while the native heterotrophic bacteria were not affected. This suggests a selective bactericidal effect of CuNZ on *A. baumannii*.

The addition of 1 wt% of AgNZ (Figure 1) resulted in complete removal within 1 h of contact. The same result was achieved with 0.1 wt% of AgNZ. Although this effect was accompanied by a statistically significant reduction of heterotrophic bacteria, their count was still high (5.2±0.2 log CFU/g) after 24 h of contact.

### *Remediation of red palaeosol contaminated with* A. baumannii

In red palaeosol supplemented with 1 wt% of NZ (Figure 2) *A. baumannii* count dropped significantly compared to baseline, but viable cells were detected throughout the four months of monitoring (2.2 log CFU/g). The bacteria did not multiply, probably due to low soil organic carbon content (0.215 wt%). An earlier study (11) showed that *A. baumannii* survived for a long time (150 days) in spring water with a pH of 8.1, which is very close to the pH of our red palaeosol samples (8.43±0.14). As moisture was kept to the maximum water holding capacity, desiccation can be eliminated as the cause of *A. baumannii* count drop. Anaerobic conditions can also be excluded, as the soil layer in glasses was not thicker than 3 cm and was regularly stirred. Rises and falls in *A. baumannii* counts observed from day 68 on could be explained by the “bust and boom” survival strategy, where weak cells die to provide sustenance for the remaining cells (9). However, the difference between the 32 % survival in red palaeosol in this study and >90 % survival in spring water in our earlier study (11) suggests that nutrient deprivation is not the only cause of lower *A. baumannii* survival in red palaeosol. Perhaps it was the presence of Fe in wet red palaeosol that contributed to the drop in *A. baumannii* count *via* oxidative stress (20).

The difference in *A. baumannii* survival between red palaeosol (four months) and *terra rossa* (seven days), both treated with NZ, is clearly owed to their difference in soil pH (8.43 *vs* 5.40, respectively), which suggests that priority in soil treatment should be given to slightly alkaline soils such as red palaeosol.

The addition of 1 wt% of CuNZ (Figure 2) completely removed *A. baumannii* after 14 days without affecting native heterotrophic bacteria by the end of the experiment.

The addition of 1 or 0.1 wt% of AgNZ (Figure 2) removed the entire *A. baumannii* population within 1 h. Heterotrophic bacterial count dropped significantly but was still 4.3±0.2 log CFU/g after 24 h of monitoring.

Considering the long survival of *A. baumannii* in red palaeosol (more than four months), we feel that the promising activity of AgNZ in slightly alkaline red palaeosol deserves more attention than in acidic *terra rossa*. AgNZ particles are bactericidal in direct contact with bacteria in soil and through leached Ag cations (14, 21). However, this antibacterial activity will depend on soil chemistry and the distribution of AgNZ particles and their contact with bacteria in soil. In our leaching test with red palaeosol 0.038 mg/L of Ag leached from 0.1 wt% (1 mg/g) of AgNZ added to the soil. This concentration of leached Ag was enough to eliminate 6.8 log CFU/g of *A. baumannii*.

Table 1 shows native heterotrophic bacteria identified in red palaeosol before and after treatment with NZ, CuNZ, or AgNZ. Treatment with NZ and CuNZ left both spore-forming (*Bacillus, Brevibacillus*, *Paenibacillus*, *Streptomyces)* and nonspore-forming genera (*Arthrobacter, Burkholderia, Pseudomonas*) alive, while only sporogenic isolates survived the treatment with AgNZ.

**Table 1:** Native soil heterotrophic bacterial isolates determined with MALDI-TOF MS in fresh red palaeosol before and at the end of treatment with 1 wt% of NZ or CuNZ or AgNZ

Isolate	Fresh soil	NZ	CuNZ	AgNZ
*Arthrobacter* sp.	6	1	1	
*Arthrobacter oxydans*		4	1	
*Arthrobacter scleromae*		1		
*Arthrobacter polychromogenes*		1		
*Bacillus sp.	2			6
*Bacillus cereus				2
*Bacillus megaterium				1
*Bacillus thuringiensis				1
*Brevibacillus sp.				1
*Burkholderia* sp.	1			
*Burkholderia caledonica*	1			
*Paenibacillus sp.	2			2
*Pseudomonas* sp.	1			
*Streptomyces sp.	2			
*Streptomyces chartreusis		1	1	
*Variovorax* sp.			1	

* spore-formers

Ag cations exert bactericidal effects by blocking the enzymatic machinery of bacterial cells (22). There are concerns about *A. baumannii* surviving in hostile environments for a long time by going dormant, that is, by retaining their viability but not culturability, which may affect cell counting. A recent study (10) has showed that *A. baumannii* could lose culturability if kept at 37 °C for a long time. However, this phenomenon of cell dormancy did not occur at 20 °C. Our experiments simulated environmental temperature of 25 °C, and AgNZ showed its effects quickly, within 1 h, which clearly excludes the possibility that the cells went dormant (non-culturable) but remained viable.

With its almost instant bactericidal effect against *A. baumannii* at very low concentrations, AgNZ could be applied in contaminated soils without negative impact on native bacterial populations. Furthermore, Ag cations stick to negatively charged soil particles (23), which minimises the possibility of their further leaching from soil, migration into groundwater, and toxic effects in the environment.

## Conclusions

The addition of AgNZ to soil resulted in removal of 6.8 log CFU/g of viable *A. baumannii* within 1 h of contact. AgNZ particles in the mass fraction of 0.1wt% (1 g per 1 kg of soil) could easily be dispersed onto soil contaminated with *A. baumannii*. Since AgNZ is prepared with a simple modification of cost-effective and environmentally friendly natural zeolite, it is a promising material for the remediation of soils contaminated with pandrug-resistant *A. baumannii*.
